# Effects of Long-Term Proton Pump Inhibitor Use on Serum Electrolytes and Vitamin Levels: A Quasi-experimental Study in Pakistan

**DOI:** 10.7759/cureus.82102

**Published:** 2025-04-11

**Authors:** Adil Khan, Mehmood Hussain, Ayaz Ahmed, Namra Nazir, Muhammad Khan Malik, Noshaba Saeed

**Affiliations:** 1 Family Medicine, Pak Emirates Military Hospital, Rawalpindi, PAK; 2 Internal Medicine, PAF Hospital, Mushaf, Sargodha, PAK; 3 Internal Medicine, Fazaia Postgraduate Medical Institute, Islamabad, PAK; 4 Medicine, PAF Hospital, Mushaf, Sargodha, PAK; 5 Internal Medicine, DHQ Teaching Hospital Sargodha, Sargodha, PAK

**Keywords:** electrolyte disturbances, hypomagnesemia, micro-nutrient, proton-pump inhibitors (ppi), vitamin b12 deficiency

## Abstract

Objective

The study aims to investigate the effects of long-term proton pump inhibitor (PPI) use on various micronutrients and electrolytes as compared to other acid-suppressive therapies in the South Asian population.

Study design and setting

This quasi-experimental study was conducted at the Department of Family Medicine in a a tertiary care setup in Rawalpindi, Pakistan from January 2022 to March 2023.

Materials and methods

Forty-nine patients with known gastric acid secretion-related disorders were randomized in two groups. The PPI group was treated with oral omeprazole, while the non-PPI group were given sucralfate and famotidine. Patients were followed up for a period of 12 months and levels of sodium, potassium, calcium, magnesium, phosphate, iron, vitamin B12, folate and vitamin D were checked using blood samples at baseline, three-, six-, and 12-month duration.

Results

The results showed that patients in the PPI group had significantly lower levels of magnesium (1.46 ± 0.15 mEq/L vs. 1.70 ± 0.14 mEq/L, p<0.01), calcium (8.96 ± 0.42 mg/dL vs. 9.50 ± 0.48 mg/dL respectively, p<0.01), and vitamin B12 (329.5 ± 134.7 pg/mL vs. 462.30 ± 193.7 pg/mL, p=0.009) as compared to non-PPI group at 12-month duration. Levels of other electrolytes and minerals did not show significant differences amongst both groups.

Conclusion

The study indicates that long-term PPI use is associated with lower levels of magnesium, calcium and vitamin B12 levels. Therefore, healthcare providers should consider monitoring these micronutrient levels in patients on long-term PPI therapy to prevent potential nutritional deficiencies.

## Introduction

Proton pump inhibitors (PPIs) are a class of medications that inhibit acid secretion in the stomach; therefore, they are used in the management of acid-related disorders, including peptic ulcer disease (PUD), gastroesophageal reflux disease (GERD) or Zollinger-Ellison syndrome [1]. They act by inhibiting the H^+^/K^+^ ATPase pump in gastric parietal cells, which is responsible for acid secretion [1,2]. By blocking this step of acid secretion, PPIs cause a reduction in the amount of gastric acid produced thus raising intra-gastric pH [3].

PPIs are among the most widely prescribed medications globally, and omeprazole is the most frequently prescribed PPI, ranking sixth among the most commonly prescribed drugs in the United States [4]. Despite their widespread use, studies suggest that a significant proportion of patients may be appropriately prescribed PPIs. In fact, estimates suggest that 30% to 50% of patients may receive PPIs inappropriately, either due to excessive treatment duration, inappropriate dosage, or inappropriate indications [5,6].

Although PPIs are generally well tolerated, this issue is a cause for concern because inappropriate use of PPIs can lead to unnecessary healthcare costs, potential drug interactions, and increased risk of adverse effects. Potential adverse effects of PPI use include increased fracture risk, electrolyte abnormalities such as hypocalcemia and hypomagnesemia, increased risk of infections, anaemia secondary to both iron and vitamin B12 malabsorption, *Clostridium difficile* infection, and increased cardiovascular risk among others [7].

The mechanism of hypomagnesemia secondary to long-term PPI use is not exactly known but is thought to be secondary to defective absorption through active or passive uptake channels or increased gut losses [7,8]. Hypophosphatemia and hyponatremia have also been described with injudicious use of PPIs especially in the elderly population [9].

Long-term PPI use is also linked with iron deficiency. Higher gastric pH is theorized to impair the absorption of non-heme iron by reducing the conversion of less absorbable ferric ions (Fe^3+^) to more absorbable ferrous ions (Fe^2+^) [10]. Few studies have revealed lower absorption of protein-bound vitamin B12 associated with PPI use and thus increased risk of vitamin B12 deficiency [8]. Some experiments have shown that gastric pH >4 results in an unstable form of vitamin C and is hydrolyzed to an ineffective and unabsorbable form resulting in its potential deficiency [11].

Malnutrition is a significant public health concern in Pakistan, with a high prevalence of undernourishment reported in many households. According to a United Nations International Children's Emergency Fund (UNICEF) report in 2017, over 40% of Pakistani households consume fewer calories than the recommended daily intake [12]. Consequently, borderline deficiencies of micronutrients are prevalent in the population. PPIs are widely prescribed and have the potential to exacerbate pre-existing nutritional deficiencies.

Therefore, the present study aims to investigate the practical effects of long-term PPI use as compared to other acid-suppressive therapies when clinically indicated and assess their effects on the levels of various micronutrients and electrolytes. The findings of this study may provide valuable insights into the potential nutritional implications of long-term PPI use.

## Materials and methods

This study was conducted as a quasi-experimental trial in the Department of Family Medicine at a tertiary care setup in Rawalpindi, Pakistan. It was a non-sponsored study and was conducted from 1 January 2022 to 31 March 2023 after approval from the ethical review board of the same hospital. The trial was registered in the Iranian Registry of Clinical Trials with ID 81437 (IRCT20240505061656N1).

Sample size estimation was performed using the Epi-Info (Centers for Disease Control and Prevention (CDC), Atlanta, USA) with a 95% confidence interval and 80% study power, based on Pasina et al. as the reference study [13]. This study revealed a general prevalence of hypomagnesemia at 14.1% and an adjusted hazard ratio of 4.31 when comparing the prevalence of hypomagnesemia in patients on PPIs as compared to those who are not on this treatment. A target sample size of 43 was calculated. We aimed to target 60 patients in our sample (30 in each group) in order to achieve better results as well as counter failure to follow-up.

Inclusion criteria

Participants included in this study were adults ≥18 years of age, had an indication for long-term PPI therapy such as GERD, chronic gastritis or Zollinger-Ellison syndrome and were willing to participate in this study.

Exclusion criteria

Patients with PUD secondary to *Helicobacter pylori*, medical conditions causing malabsorption (such as celiac disease, chronic pancreatitis, gut resection, inflammatory bowel disease, and chronic diarrhoea), and those with pre-existing electrolyte or vitamin deficiencies requiring intervention were excluded from this study.

A convenience sampling technique was used to induct patients visiting family medicine clinics who fulfilled inclusion and exclusion criteria. Patients were randomly divided into two groups at 1:1 using a computerized balloting method. Neither the participants nor the investigators were blinded. Baseline data was collected from individuals including their age, gender, educational status, previous comorbid conditions and current symptoms. A stool sample was taken to rule out active *H. pylori* infection.

Blood samples were taken to check levels of sodium, potassium, magnesium, calcium, phosphate, iron, vitamin B12, folate and vitamin D levels. Assays for micronutrient analysis were performed using the Roche Cobas® Pure system (Roche Diagnostics International Ltd., Rotkreuz, Switzerland). Sodium and potassium were measured using ion-selective electrodes. Immunoassay methods were employed for analyzing vitamin B12, folate, and vitamin D levels, while the remaining parameters were assessed through photometry.

The PPI group was started on oral omeprazole 20 mg to 40 mg per day while the control group were given either famotidine or sucralfate. Patients were followed up for a period of 12 months. Repeat samples were taken at three-, six-, and 12-month duration. Patients were also interviewed for subjective side effects and the effectiveness of the given treatment. Individuals were advised not to take nutritional/vitamin/iron supplements during the course of the study. Patients failing to follow up were labelled as failure to follow-up and their data was removed from statistical analysis.

**Figure 1:**
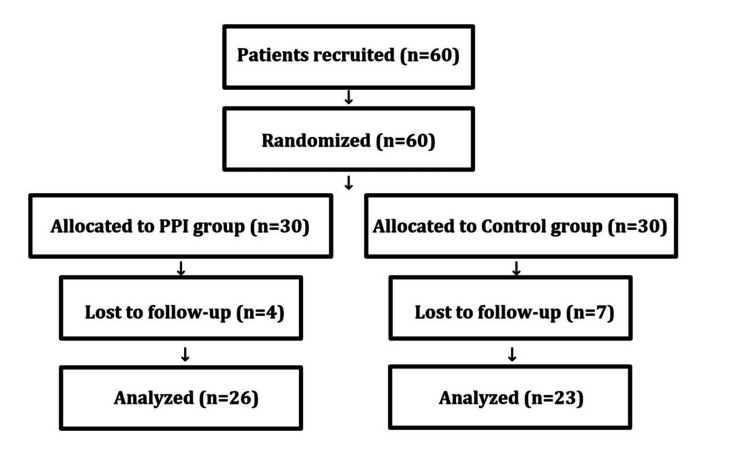
CONSORT flow diagram CONSORT: Consolidated Standards of Reporting Trials; PPI: proton pump inhibitor

In this study, we initially included 60 individuals, with 30 participants in each group. However, due to follow-up losses during the course of the trial, our final analysis was conducted on 49 individuals, as shown in the Consolidated Standards of Reporting Trials (CONSORT) flow diagram (23 in the control group and 26 in the PPI group) (Figure 1).

Data was collected and analyzed in Microsoft Excel (Microsoft® Corp., Redmond, WA, USA) and Statistical Package for the Social Sciences (IBM SPSS Statistics for Windows, IBM Corp., Version 25.0, Armonk, NY). Statistical analysis was done in order to determine any significant changes in levels of the abovementioned electrolytes, minerals and vitamins between the two groups. Student's t-test was used to compare the two groups and t-value was calculated. A difference in p-value <0.05 was considered significant.

## Results

The mean age of our study population was 47.92 ± 13.46 years, with an even distribution of gender (25 males and 24 females). The most common comorbid conditions observed in our study population were diabetes (n=21, 42.86%) and hypertension (n=18, 36.73%) (Table 1).

**Table 1:** Baseline characteristics of study population A p-value <0.05 was considered statistically significant. The Student’s t-test was used to compare the two groups. IHD: ischemic heart disease; OAD: obstructive airway disease

		Population (n=49)	Treatment group (n=26)	Control group (n=23)	t-value	p-value
Age, mean±SD		47.918 ± 13.464	47.77 ± 13.36	48.09 ± 13.88	-0.082	0.935
Gender	Male, n (%)	25 (51.02)	13 (50)	12 (52.17)	0.149	0.882
	Female, n (%)	24 (48.98)	13 (50)	11 (47.83)		
Education	Upto primary, n (%)	16 (32.65)	9 (34.62)	7 (30.43)	0.597	0.551
	Upto matriculation, n (%)	10 (20.41)	3 (11.54)	7 (30.43)		
	Upto intermediate, n (%)	12 (24.49)	7 (26.92)	5 (21.74)		
	More than intermediate, n (%)	11 (22.45)	7 (26.92)	4 (17.39)		
Comorbid	Diabetes, n (%)	21 (42.86)	10 (38.46)	11 (47.83)	-0.649	0.519
	Hypertension, n (%)	18 (36.73)	10 (38.46)	8 (34.78)	0.261	0.795
	IHD, n (%)	10 (20.41)	4 (15.38)	6 (26.09)	-0.905	0.370
	Thyroid disease, n (%)	8 (16.33)	4 (15.38)	4 (17.39)	-0.185	0.854
	OAD, n (%)	13 (26.53)	6 (23.08)	7 (30.43)	-0.569	0.570
	Others, n (%)	15 (30.61)	5 (19.23)	7 (30.43)	-0.890	0.378
Sodium (mEq/L), mean ± SD		139 ± 3.45	139.42 ± 3.71	138.52 ± 3.15	0.920	0.362
Potassium (mEq/L), mean ± SD		4.19 ± 0.53	4.20 ± 0.49	4.17 ± 0.59	0.142	0.887
Magnesium (mEq/L), mean ± SD		1.67 ± 0.24	1.65 ± 0.26	1.69 ± 0.21	-0.595	0.555
Calcium (mg/dL), mean ± SD		9.42 ± 0.56	9.41 ± 0.51	9.43 ± 0.63	-0.141	0.889
Phosphate (mg/dL), mean ± SD		3.66 ± 0.38	3.65 ± 0.42	3.68 ± 0.33	-0.218	0.828
Iron (ug/dL), mean ± SD		108.53 ± 51.94	108.85 ± 49.69	108.17 ± 55.49	0.044	0.965
Vitamin B12 (pg/mL), mean ± SD		440.08 ± 214.71	417.58 ± 209.07	465.52 ± 222.79	-0.774	0.443
Folate (ng/mL), mean ± SD		10.49 ± 4.55	10.08 ± 4.25	10.95 ± 4.92	-0.662	0.511
Vitamin D (nmoL/L), mean ± SD		39.98 ± 12.16	40.12 ± 13.33	39.83 ± 10.99	0.083	0.934

Our baseline characteristics analysis showed no significant differences between age, gender, educational status, and comorbid conditions among the two groups. Additionally, at baseline, there were no significant differences in levels of sodium, potassium, calcium, magnesium, phosphate, iron, folate, vitamin B12, and vitamin D between the two groups. The baseline characteristics of the study population and two groups are described in Table 1 in detail.

During the study period, levels of electrolytes and micronutrients remained relatively stable in the control group. However, in the PPI group, we observed significant changes in levels of magnesium, calcium, iron, and vitamin B12. Specifically, we found that levels of magnesium, calcium, iron and vitamin B12 were significantly lower, while levels of vitamin D were significantly higher compared to baseline. Details of the comparison are given in Table 2.

**Table 2:** Baseline versus 12-month serum levels of magnesium, calcium, iron and vitamin B12 in the PPI group (n=26) A p-value < 0.05 was considered significant. A paired t-test was used to compare baseline and 12-month serum levels within the PPI group. PPI: proton pump inhibitor

	Level at baseline (mean±SD)	Level at 12-month (mean±SD)	t-value	p-value	Mean difference	95% lower confidence interval	95% upper confidence interval
Magnesium (mEq/L)	1.64 ± 0.263	1.46 ± 0.152	4.239	<0.001	0.191	0.068	0.313
Calcium (mg/dL)	9.41 ± 0.514	8.96 ± 0.416	3.790	<0.001	0.450	0.183	0.717
Iron (ug/dL)	108.9 ± 49.6	89.4 ± 37.3	4.395	<0.001	19.42	-5.67	44.51
Vitamin B12 (pg/mL)	417.6 ± 209	329.5 ± 135	3.859	<0.001	88.04	-12.41	188.48

When comparing the two groups, we found significantly lower levels of magnesium in the PPI group at 6- and 12-month periods compared to controls (p=0.007 and p<0.001, respectively). Additionally, serum levels of calcium were significantly lower in the PPI group at 6- and 12-month periods compared to controls (p=0.016 and p<0.001, respectively). Levels of vitamin B12 were also significantly lower in the PPI group at 12 months compared to controls (p=0.008), while levels of vitamin D were significantly higher in the PPI group at 12 months compared to controls (p=0.03). Finally, we found no significant alterations in levels of sodium, potassium, phosphate, iron, and folate between the PPI and control groups at any time during the course of the study. Details of this analysis are given in Table 3.

**Table 3:** Comparison of electrolyte levels between the PPI and non-PPI groups was conducted at various time intervals A p-value <0.05 was considered statistically significant. The Student’s t-test was used to compare the two groups. PPI: proton pump inhibitor

		PPI group, Mean±SD (n=26)	Control group, Mean±SD (n=23)	t-value	p-value
Sodium (mEq/L)	Baseline	139.42 ± 3.71	138.52 ± 3.15	0.920	0.362
	3 months	138.62 ± 3.44	139.83 ± 3.07	-1.302	0.199
	6 months	139.19 ± 2.70	139.00 ± 2.89	0.240	0.812
	12 months	138.62 ± 3.81	139.43 ± 2.83	-0.862	0.393
Potassium (mEq/L)	Baseline	4.20 ± 0.49	4.17 ± 0.59	0.142	0.887
	3 months	4.12 ± 0.36	4.10 ± 0.35	0.146	0.885
	6 months	4.11 ± 0.37	4.16 ± 0.30	-0.552	0.583
	12 months	4.06 ± 0.37	4.11 ± 0.35	-0.534	0.596
Magnesium (mEq/L)	Baseline	1.65 ± 0.26	1.69 ± 0.21	-0.595	0.555
	3 months	1.59 ± 0.24	1.69 ± 0.17	-1.672	0.101
	6 months	1.54 ± 0.22	1.69 ± 0.14	-2.852	0.007
	12 months	1.46 ± 0.15	1.70 ± 0.14	-5.769	<0.001
Calcium (mg/dL)	Baseline	9.41 ± 0.51	9.43 ± 0.63	-0.141	0.889
	3 months	9.32 ± 0.48	9.42 ± 0.37	-0.773	0.444
	6 months	9.17 ± 0.41	9.47 ± 0.41	-2.500	0.016
	12 months	8.96 ± 0.42	9.50 ± 0.48	-4.180	<0.001
Phosphate (mg/dL)	Baseline	3.65 ± 0.42	3.68 ± 0.33	-0.218	0.828
	3 months	3.60 ± 0.36	3.69 ± 0.35	-0.909	0.368
	6 months	3.63 ± 0.32	3.66 ± 0.29	-0.399	0.692
	12 months	3.55 ± 0.35	3.62 ± 0.27	-0.809	0.423
Iron (ug/dL)	Baseline	108.85 ± 49.69	108.17 ± 55.49	0.044	0.965
	3 months	103.31 ± 44.48	106.43 ± 49.37	-0.232	0.818
	6 months	100.85 ± 44.96	104.87 ± 42.76	-0.321	0.750
	12 months	89.42 ± 37.27	104.74 ± 38.34	-1.414	0.164
Vitamin B12 (pg/mL)	Baseline	417.58 ± 209.07	465.52 ± 222.79	-0.774	0.443
	3 months	393.15 ± 167.02	462.22 ± 213.51	-1.250	0.218
	6 months	371.88 ± 153.87	465.43 ± 203.80	-1.795	0.080
	12 months	329.54 ± 134.67	462.30 ± 193.66	-2.752	0.009
Folate (ng/mL)	Baseline	10.08 ± 4.25	10.95 ± 4.92	-0.662	0.511
	3 months	10.32 ± 3.65	10.96 ± 4.07	-0.581	0.564
	6 months	10.23 ± 3.96	10.76 ± 3.82	-0.480	0.634
	12 months	9.65 ± 3.34	10.69 ± 3.49	-1.055	0.297
Vitamin D (nmoL/L)	Baseline	40.12 ± 13.33	39.83 ± 10.99	0.083	0.934
	3 months	42.96 ± 12.91	39.13 ± 7.78	1.274	0.210
	6 months	45.92 ± 12.72	41.48 ± 8.17	1.471	0.149
	12 months	48.31 ± 12.44	42.22 ± 7.22	2.124	0.040

## Discussion

Gastric acid is an important digestive instrument and plays a vital role in the absorption and homeostasis of various minerals and micronutrients such as magnesium, calcium, iron, cobalamin and ascorbic acid. Thus, PPIs can potentially cause deficiencies of these substances by reducing stomach acid secretion [14]. This unwanted effect of PPIs can lead to hematopoietic disturbances leading to micro- or macrocytic anaemia, electrolyte imbalance resulting in paresthesia, muscle cramps or even life-threatening arrhythmias, or effect bone formation resulting in osteoporosis among other side effects [14].

To better understand the true impact of these potential deficiencies, this study was designed to compare patients on long-term PPIs with other potential treatment options for stomach acid-related disorders. Patients were tested for potential deficiencies of sodium, potassium, calcium, magnesium, phosphate, iron, vitamin B12, folate and vitamin D and results have indicated a significant reduction in levels of magnesium, calcium iron and vitamin B12 in patients on long-term PPI therapy. When compared with patients on other therapies, levels of magnesium, calcium and vitamin B12 were significantly lower in the PPI group as compared to patients on other treatment options.

Low magnesium levels can decrease parathyroid hormone secretion, leading to both hypocalcemia and altered bone metabolism [7,8]. Lower parathyroid hormone concentration is linked to increased fracture risk seen with chronic PPI use. Low magnesium levels can also interfere with renal potassium reabsorption leading to hypokalemia [7,8].

Results of this study indicated that there was a significant reduction in serum levels of magnesium after 12 months in the PPI group (mean difference: 0.191 mEq/L, 95% CI: 0.068-0.313, p<0.001). Furthermore, the serum magnesium levels were significantly lower in the PPI group when compared to the non-PPI group (1.70 ± 0.14 mEq/L vs. 1.46 ± 0.15 mEq/L, p<0.01) after 12 months of treatment. Recart et al. (2021) also observed similar findings through a retrospective study of individuals receiving long-term PPI therapy (defined as more than six months). Specifically, 36% of patients on long-term PPI showed evidence of hypomagnesemia (magnesium level <1.3 mEq/dL) with a mean magnesium level of 1.56 mEq/L [15].

Likewise, a meta-analysis of 16 studies published by Srinutta et al. (2019) in November of that year demonstrated an adjusted odds ratio of 1.71 (95% CI: 1.33-2.19; p<0.001) for the risk of hypomagnesemia with chronic PPI use. This study also identified higher odds of hypomagnesemia with higher PPI doses as compared to lower doses (OR 2.13; 95% CI: 1.26-3.59; p=0.005) [16]. Lower levels of serum magnesium have been associated with life-threatening arrhythmia, Torsades de Pointes and it was observed by Lazzerini et al. (2018), that in unselected patients with this arrhythmia, more than 50% had evidence of concurrent PPI use [17].

The present study found a significant decrease in serum total calcium levels in patients on long-term PPI therapy, with a mean difference of 0.450 mg/dL at the end of the 12-month treatment period (CI: 0.183-0.717, p<0.01). Furthermore, there was a significant difference in serum calcium levels at the end of the 12-month treatment period between patients on PPI therapy and those not on PPI therapy, with the former group exhibiting lower levels (8.96 ± 0.42 mg/dL vs. 9.50 ± 0.48 mg/dL respectively, p<0.01). While there is a link between PPI use and osteoporotic fracture, the use of PPI has been inconsistently linked with hypocalcemia [8,18].

The mechanism of hypocalcemia has been theorized as secondary to a reduction in calcium absorption or an indirect result of hypomagnesemia. Recart et al. (2021) observed that in patients on long-term PPIs, hypocalcemia was seen more frequently in patients with hypomagnesemia as compared to those with normomagnesemia (57% vs. 38.7%, p<0.05) [15]. Similarly, O’Connell et al. (2005) observed a reduction in calcium absorption by up to 40% in females taking omeprazole for only 14 days [19]. This reduction in absorption is theorized to induce parathyroid hormone release which in an attempt to keep serum calcium levels constant can lead to osteoporosis.

Serum vitamin B12 levels were significantly lower at the end of the 12-month period in PPI users with a mean reduction of 88.04 pg/mL (95% CI: -12.41 to 188.48, p<0.01). In comparison to the non-PPI group, the PPI group had significantly lower serum vitamin B12 levels (462.30 ± 193.7 pg/mL vs. 329.5 ± 134.7 pg/mL respectively, p=0.009). This reduction is theorized due to reduced absorption. Gastric acid is required to convert pepsinogen to its active form pepsin. Pepsin is necessary for protein metabolism and the release of vitamin B12 from large proteins, thus reducing its absorption.

A meta-analysis of 25 studies published in 2023 has also revealed similar findings. Odds of vitamin B12 deficiency were higher in patients on PPI as compared to those not on PPI (OR 1.42, 95% CI: 1.16-1.73) [20]. Similarly, a systematic review on the effects of PPIs on vitamin B12 metabolism, published in 2022, has provided indirect evidence of vitamin B12 deficiency by studying markers of its deficiency such as elevated methyl-malonic acid or rise in mean corpuscular volume (MCV) [21].

Reduction in serum iron levels was also observed in patients on long-term PPIs. The mean reduction in serum iron levels was 19.42 ug/dL (CI: -5.67 to 44.51 ug/dL, p<0.01). Although there was an observed difference in 12-month iron levels between PPI and non-PPI groups (89.4 ± 37.27 ug/dL vs. 104.74 ± 38.34 ug/dL, p=0.164), the difference was not significant. Stomach acid helps iron absorption by converting it from less absorbable ferric iron (Fe^3+^) to more absorbable ferrous iron (Fe^2+^). Thus, reduction in stomach acid through any means is theorized to impair its absorption. Similar results have been seen in a case-control study published in 2018 with a sample size of 27,897 individuals. In this study, the odds ratio of iron deficiency was 3.60 (95% CI: 3.32-3.91) between PPI users and non-users [22].

This study did not see any significant reduction in sodium, potassium, phosphate, folate or vitamin D levels after 12-month PPI use. Few studies have noted altered absorption of vitamin D; however, results have not been consistent. Similarly, both hyponatremia and hypokalemia have been reported in case reports; however, few small-scale studies have been unable to find this correlation [23].

Even though the study was done on a smaller sample size, it did detect a potential adverse effect of excessive PPI use that includes deficiencies in essential micronutrients like magnesium, calcium, iron, and vitamin B12. This raises the question of whether these deficiencies will lead to significant clinical outcomes, highlighting the need for further research to explore the long-term health implications.

This study is limited by its small sample size and short follow-up period of 12 months. Furthermore, confounding factors such as undiagnosed malnutrition and other medications that may influence the absorption of micronutrients were not specifically accounted for in this study; however, these factors were addressed through randomization. Further research through larger, multicenter, randomized controlled trials and population-based studies may be needed to better understand the effects of PPIs on electrolytes and micronutrients.

## Conclusions

The study aimed to investigate the potential impact of long-term PPI use on serum levels of minerals and micronutrients. The results suggest that long-term PPI use can lead to deficiencies of magnesium, calcium, iron, and vitamin B12. The reduction in serum levels of these nutrients can lead to various side effects, including hematopoietic disturbances, electrolyte imbalance, osteoporosis, and life-threatening arrhythmias.
